# Meningeal Signs and Cerebellar Involvement in Scrub Typhus: A Case Report

**DOI:** 10.7759/cureus.25708

**Published:** 2022-06-07

**Authors:** Sadikshya Bhandari, Samikshya Bhandari, Kushal Gautam, Roshan Jha, Sijuka Devkota

**Affiliations:** 1 Internal Medicine, Dhulikhel Hospital, Dhulikhel, NPL; 2 Internal Medicine, Nepal Medical College Teaching Hospital, Kathmandu, NPL; 3 Paediatric Research Unit, Patan Academy of Health Sciences, Kathmandu, NPL; 4 General Medicine, P.T. (Purna Tunga) Birta City Hospital, Jhapa, NPL

**Keywords:** neglected disease, meningeal irritation, cerebellar signs, scrub typhus, case report

## Abstract

Scrub typhus is an arthropod-borne fever that follows the bite of the larval form of Leptotrombidium mite carrying *Orientia tsutsugamushi*. It remains a serious health problem in the Asia-Pacific region. While it commonly presents as an undifferentiated fever with chills and an eschar, complications like pneumonitis, acute respiratory distress syndrome, disseminated intravascular coagulation, and meningoencephalitis may cause scrub typhus to be fatal. However, regardless of the dramatic presentation, treatment with antibiotics, preferably doxycycline or even azithromycin, is effective in recovery.

In this case report, we present a case of meningitis and cerebellar involvement in an adolescent with positive scrub typhus serology in the absence of an eschar. This brought forward a diagnostic delay as other infections including tuberculosis were considered before scrub typhus due to unusual presenting symptoms and the lack of an eschar. Thus, in cases like these, it becomes imperative to be aware of the unusual manifestations to initiate antibiotics on time and prevent further complications.

## Introduction

Scrub typhus is caused by an arthropod-borne, gram-negative, obligate intracellular bacillus, *Orientia tsutsugamushi* (*O. tsutsugamushi*), which spreads via the bite of the larval stage of the Leptotrombidium mite, widely known as chiggers [1,2]. 

Scrub typhus remains one of the common causes of acute undifferentiated febrile cases. It is also a common cause of hospitalization in rural areas of Asia, the North of Australia, and many islands of the Pacific Ocean with one billion people at risk of infection [1,2]. 

Typical manifestations of the infection include non-specific flu-like symptoms: fever, rash, headache, myalgia, cough, generalized lymphadenopathy, nausea, vomiting, and abdominal pain [3]. Eschar, if present, at the bite site is diagnostic [3]. However, the non-specific clinical presentation of scrub typhus with involvement of the cardiovascular system, respiratory or gastrointestinal symptoms, renal or endocrine involvement, hematological involvement as well as neuropsychiatric involvement has resulted in delayed treatment with antibiotics. This has increased the risk of case fatality in patients with atypical presentations [1,3]. 

Here, we describe the case of an adolescent who presented with fever associated with signs of meningeal and cerebellar involvement, in the absence of an eschar, with serology positive for scrub typhus. 

## Case presentation

A 14-year-old male, with no significant past medical history, was referred to the emergency department with a fever for six days and a maximum documented temperature of 101°F. He had no rash. Fever was not associated with chills, rigor, or sweating. However, the patient had complaints of slurring of speech. He, however, had no problems in comprehension, understanding, or production of speech. 

Following the development of fever, he also had a headache for five days, which was generalized with no aggravating factors. It was relieved by oral analgesics. He also had vomiting, around 5-6 bouts, on the day before the presentation, which was watery and projectile in nature.

He had no history of similar symptoms in the past. He had no history of any illness in the past and had not used any medications. He had been vaccinated at birth against tuberculosis. There was also no history of similar illness in any of his family members. 

On examination, the patient was well oriented to person, place, and time but seemed agitated. His Glasgow coma scale (GCS) score was 15. His temperature was 101°F. His pulse rate, blood pressure, and respiratory rate were normal as per his age and his oxygen saturation was 97% on room air. On examination, his bilateral inguinal, submandibular, as well as occipital lymph nodes were palpable but non-tender. There were no overlying skin changes. Pallor, icterus, cyanosis, clubbing, and dehydration were absent. 

On neurological examination, the patient had neck rigidity with positive Kernig’s and Brudzinski’s signs. His speech was slurred with normal comprehension and repetition. Cranial nerves were intact with normal smell, vision, hearing, facial sensations, and taste and he had no difficulty swallowing. Sensory examination showed normal pain, touch, and temperature sensations with normal position, vibration, and discriminative sensations bilaterally. Plantar reflex was bilaterally downgoing. Lower limb reflexes were 1+ (diminished) in his knees and ankles bilaterally. However, jerks were normal in both his upper limbs. He had normal tone, bulk, and power bilaterally in both upper and lower limbs. However, a cerebellar examination showed bilateral gaze-evoked nystagmus. He had an ataxic gait and had to be supported while walking. The patient also had dysmetria. 

His abdomen was soft and tender. The liver and spleen were not palpable. On chest auscultation, there was equal air entry bilaterally without any added sounds. 

An ophthalmological examination was done, which showed bilateral papilledema with the normal macula and hyperemic optic disc. Lumbar puncture (LP) could not be done due to signs of raised intracranial pressure (ICP). Guarded LP was not available. As the patient had been referred from another tertiary center, he had already been given intravenous (IV) mannitol and IV dexamethasone in its emergency room.

As seen in Table 1, his investigations following the use of mannitol and dexamethasone showed decreased platelet count while his hemoglobin and white blood count were normal. A low level of sodium was seen with normal potassium levels. C-reactive protein was also raised. The rest of the investigations including urine analysis were normal. Routine serology for HIV and hepatitis B was non-reactive. 

**Table 1:** Investigation findings on admission CRP, C-reactive protein.

Parameters	Findings	Normal value
Total leukocyte count (/µL)	10500	4000-11000
Neutrophil %	73	40-60
Lymphocytes %	26	20-40
Hemoglobin (g/dL)	14.1	11.5-15
Platelets (/µL)	122000	150000-450000
Sodium (mmol/L)	128	135-145
Potassium (mmol/L)	4.4	3.7-4.7
CRP (mg/L)	37	<10
Urea (mg/dL)	33	14-23
Calcium (mg/dL)	8.7	8-10
Creatinine (mg/dL)	0.7	0.7-1.3
Random blood sugar (mmol/L)	119	<140
Blood culture	No organism growth seen	

He was then admitted to the inpatient ward with a provisional diagnosis of meningitis with generalized lymphadenopathy and was started on IV ceftriaxone 2 g and mannitol 50 mL along with hypertonic saline. Mantoux test for tuberculosis was planned along with lymph node biopsy. Serology for scrub typhus was also sent as a part of the fever panel following a negative blood culture. Computed tomography of the head was done, which showed no abnormalities.

He was then transferred to the pediatric intensive care unit (PICU) due to the need for monitoring for his raised ICP. He was hemodynamically stable for 48 hours and was then shifted back to the pediatric ward. 

On the fifth day of admission, serology for scrub typhus via rapid immunoglobulin M (IgM) antibody came positive. Hence, he was started on IV chloramphenicol 1 g which was continued for 14 days. Following the use of chloramphenicol, in a matter of days, there was the resolution of his nystagmus as well as ataxia. He was then discharged home. 

## Discussion

Scrub typhus is one of the underdiagnosed and under-reported causes of fever which requires hospitalization [4]. Scrub typhus has a seasonal transmission with peaks in the monsoon season, in Southeast and East Asia whereas transmission is seen throughout the year in tropical and subtropical regions [4]. *O. tsutsugamushi* is the causative organism of scrub typhus. It is an obligate intracellular bacterium with unique properties. Unlike other gram-negative bacteria, it lacks lipopolysaccharides and has low levels of unclassical peptidoglycans with a unique genome with 42% of its genetic content consisting of repeat DNA sequences and mobile genetic elements that vary between strains [5,6]. Genetic variations in tumor necrosis factor-alpha and -beta are often linked to worse outcomes [4].

After initial inoculation following the bite of Leptotrombidium mite, the bacterium enters the dermal layer. Dendritic cells and activated monocytes are the target cells that lead to the dissemination of the bacterium and transfer it to the lymph nodes [4]. Bacterial adhesion to fibronectin and entry of the bacteria into host cells can result in endothelial involvement of several organs which include the brain, heart, lung, kidney, pancreas, and even the skin [7]. This disseminated vasculitis is responsible for most of the symptoms and complications of scrub typhus [8]. 

Scrub typhus most commonly presents with undifferentiated fever following an incubation period of 6-21 days [8]. Along with acute-onset fever with chills, the patient may also have headaches, vomiting, backache, myalgia, and profuse sweating [4,8]. Eschar, which appears 2-3 days before the fever, is one of the identifying features of scrub typhus; it was seen in 55% of patients in a recent study from South India [8,9]. Lymphadenopathy may also be present [8]. According to a study done from 2003 to 2011 in Laos, which included 1112 people with signs of central nervous system (CNS) infection, fever was seen in 100% of scrub typhus meningitis cases and headache in 89% [10]. Vomiting occurs in 60% of scrub typhus cases [10]. 

Complications are common after one week of illness, if untreated, which include liver or renal failure, pneumonia, acute respiratory distress syndrome, disseminated intravascular coagulation, sepsis, myocarditis, and meningoencephalitis, among others [11-13]. A complicated illness can be fatal [11]. In Nepal, in the year 2016, over 800 cases were detected, with a fatality of 1.7% [14]. In another study done in Korea, 297 patients were diagnosed with scrub typhus over six years with a mortality rate of 6.1% [15]. 

Complications in the CNS include symptoms of meningitis, encephalitis, or meningoencephalitis [11-13]. However, reported cases of meningismus in scrub typhus have been scanty with few similar cases, one of which was reported in 2020 in India, where a 42-year-old man had presented with signs of meningitis and shortness of breath. His scrub typhus IgM antibody was positive by immunochromatographic method and he was diagnosed with scrub typhus meningitis even in the absence of eschar [16]. A similar clinical picture of a febrile illness with meningitis and the absence of an eschar has been seen in our case. Another study done in India, over two years, in 37 people who had scrub typhus and presented with signs of encephalitis showed that 84% of patients had impaired consciousness and six were deeply comatose (GCS score ≤8). Twenty-two percent of patients presented with status epilepticus and focal weakness was present in 38% [17]. 

Another unusual neurological presentation as seen in this indexed case is cerebellar symptoms. Similar findings of ataxia and dysmetria were reported but with sensorineural hearing in a 53-year-old female who had a typical presentation of a febrile illness with eschar but three weeks later developed cerebellar dysfunction, lateral gaze palsy with sensorineural hearing loss [18]. Spastic dysarthria and limb ataxia were also seen in patients in a study done in South Korea with scrub typhus encephalitis [19]. 

The presence of multiple symptoms makes scrub typhus a difficult condition to diagnose, which increases fatality in cases with atypical presentations [1,3]. Indirect immunoperoxidase assay and immunofluorescence assay are considered the gold standard tests for diagnosis; however, they are not available in secondary centers. As such, molecular diagnosis via a polymerase chain reaction, most commonly immunoglobulin M capture assays, is used [11].

Regardless of the dramatic presentation of scrub typhus, it responds rapidly to antibiotics with improvement in clinical features seen as early as 48 hours with minimum sequelae [4,17]. Therefore, recognizing the unusual features and early initiation of antibiotics, even on suspicion, reduce the morbidity and mortality related to the disease [11]. 

The use of antibiotics in patients depends on their circumstances including both age and pregnancy as well as the cost and side-effect profile of the drugs, and existing local prescription guidelines [13]. Commonly used antibiotics include chloramphenicol, tetracycline, doxycycline, macrolides, quinolones, and rifampicin [4,20]. In a review published in 2017, 11 studies from Asia were included with 957 patients which concluded that doxycycline was the most frequently used antibiotic [13]. Chloramphenicol, tetracycline, and azithromycin also showed equal efficacy to doxycycline in achieving clinical cure with cure rates in all studies varying from 64% to 100% [13]. Moreover, azithromycin in comparison to doxycycline had equal efficacy in clinical cure with fewer gastrointestinal adverse events [13]. In the presented case, chloramphenicol was used for 14 days with complete resolution of symptoms. 

Similar presentations of undifferentiated fever and meningeal involvement in other tropical febrile diseases endemic to Nepal bring forward a diagnostic delay. Furthermore, in such cases with atypical presentations in the absence of an eschar, confusion can occur due to a similar presentation of tubercular meningitis which is more common in rural Asia. This leads to a delay in starting antibiotics which occurred in the indexed case where the diagnosis was made only on the fifth day of admission. As such, we as physicians have to be conscious of the varied presenting symptoms and complications of scrub typhus, and also include it in the differential diagnosis of fever. 

## Conclusions

Although scrub typhus usually presents as an acute febrile illness, its diagnosis is often delayed in the absence of an eschar because of similarities with other tropical febrile infections and due to its multiple unusual manifestations as seen in the indexed case. Awareness of these unusual clinical manifestations will help the clinician to arrive at an early diagnosis, resulting in an early administration of appropriate antibiotics and a significant decrease in the severity and fatality of the disease.
